# Potential Factors That Contribute to Post-COVID-19 Fatigue in Women

**DOI:** 10.3390/brainsci12050556

**Published:** 2022-04-26

**Authors:** Thorsten Rudroff, Craig D. Workman, Andrew D. Bryant

**Affiliations:** 1 Department of Health and Human Physiology, University of Iowa, Iowa City, IA 52242, USA; craig-workman@uiowa.edu; 2 Department of Neurology, University of Iowa Hospitals and Clinics, Iowa City, IA 52242, USA; 3 Department of Internal Medicine, University of Iowa Hospitals and Clinics, Iowa City, IA 52242, USA; andrew-d-bryant@uiowa.edu

**Keywords:** post-COVID 19, fatigue, sex differences, inflammation

## Abstract

Mortality of acute coronavirus disease (COVID-19) is higher in men than in women. On the contrary, women experience more long-term consequences of the disease, such as fatigue. In this perspective article, we proposed a model of the potential factors that might contribute to the higher incidence of post-COVID-19 fatigue in women. Specifically, psycho-physiological factors are features that might increase central factors (e.g., inflammation) and result in greater perceptions of fatigue. Furthermore, pre-existing conditions likely play a prominent role. This model offers a framework for researchers and clinicians, and future research is required to validate our proposed model and elucidate all mechanisms of the increased incidence and prevalence of post-COVID-19 fatigue in women.

## 1. Introduction

Confirmed acute coronavirus disease (COVID-19) cases have exceeded 430 million globally and 78 million in the USA [1]. Importantly, the number of patients that experience persistent symptoms during recovery is rapidly growing [2,3]. Post-COVID-19 is a disorder that appears in people with a history of probable or confirmed severe acute respiratory syndrome coronavirus 2 (SARS-CoV-2) infection, usually 3 months from the onset of COVID-19, with symptoms that last for at least 2 months and cannot be explained by an alternative diagnosis (World Health Organization (WHO)).

Fatigue is one of the primary persistent symptoms, has been reported in 44–70% of patients [4,5,6], and is independent of the severity of initial COVID-19 infection (i.e., hospitalized vs. non-hospitalized patients) [7]. According to Rudroff et al. [8], post- COVID-19 fatigue can be defined as “the decrease in physical and/or mental performance that results from changes in central, psychological, and/or peripheral factors due to the COVID-19 disease”. Thus, post-COVID-19 fatigue is dependent on both conditional and psycho-physiological factors comprising the task, environment, and physical and mental capacity of the individual and the central, psychological, and peripheral aspects of the disease. Relevantly, post-COVID-19 fatigue can persist for 6 months or even longer [9]_._

Mortality from COVID-19 is higher in men than in women; on the contrary, women are more prone to experiencing long-term consequences, such as post-COVID-19 fatigue [10,11,12]. Younger women (≤50 years) report more often than men that they do not feel “recovered,” and indicate greater disability and worse fatigue [13]. Furthermore, Sigfrid et al. [13] found that female sex was associated with pain or discomfort, anxiety, and depression symptoms. In their LONG-COVID-Exp-CM Multicenter Study, Fernández-de-las-Peñas et al. [10] also described significantly higher reports of post-COVID-19 symptoms (fatigue, pain symptoms, anxiety, depression, and poor sleep quality) in women.

The results of the above studies beg the question, “why do women have worse outcomes than men?” One reason might be initial exposure because women tend to have occupations (e.g., education) where exposure to SARS-CoV-2 might be higher [14]. Though, recent data implies that teachers do not have greater contact than other working-age populations and there is developing indications of differing host responses to SARS-CoV-2 infection [15,16]. Furthermore, because women are more likely to survive acute SARS-CoV-2 infection than men [13], the greater prevalence of post-COVID-19 fatigue in women might be biased by more women surviving acute infection. However, more research is needed to investigate sex differences across several measures of disease severity; still, such investigations would benefit from considering if the greater prevalence of symptom severity might be a function of more women surviving acute infection.

**Figure 1:**
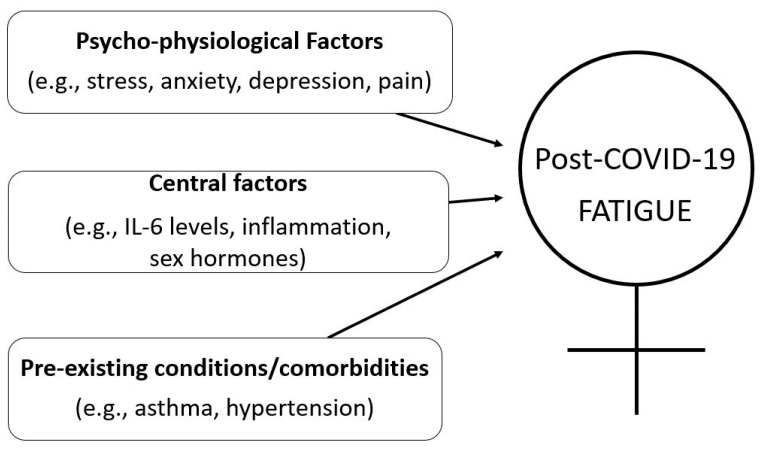
A proposed model for contributors to the increased prevalence of post-COVID-19 fatigue in women. Fatigue is greatly affected by the interactive changes in psycho-physiological factors (stress, anxiety, depression, and pain), central factors (inflammation, sex hormones), and by pre-existing conditions.

The exact causes and potential contributors to post-COVID-19 fatigue in women are currently unclear. However, based on our post-COVID-19 fatigue model [9], this perspective paper highlights factors that might provide insight into the potential reasons why women are more prone to post-COVID-19 fatigue. Specifically, stress, anxiety, depression, pain, inflammation, and pre-existing conditions are potential candidates contributing to fatigue (Figure 1). However, and importantly, the reason of fatigue cannot be reduced to a single source and other factors might also contribute to post-COVID-19 fatigue.

## 2. Factors Contributing to Post-COVID-19 Fatigue in Women

### 2.1. Stress, Anxiety, Depression, and Pain

Many actions used to fight the pandemic, such as social distancing, quarantine, and isolation, have proven effective at slowing the spread of the virus, but these might also have unintended effects that intensify fatigue in recovering COVID-19 patients [17,18,19]. COVID-19-related fatigue can concurrently appear in an environment where depression, anxiety, and stress are also prevalent [17]. Feeling stress and anxiety about the pandemic and being physically inactive in quarantine, might result in increased fatigue. Gebhard et al. [20] found that continuing neuropsychiatric symptoms were significantly higher in women compared to men, indicating that women present a higher susceptibility to long-term neurological and mental health consequences of COVID-19. Women also reported higher domestic stress levels than men [20], indicating that increased stress at home might play a significant role in post-COVID-19 fatigue. More mental health consequences, especially higher stress levels and higher levels of anxiety and depression, were also reported in women who survived the SARS epidemic in 2003 [21]. Recent work has also revealed correlations between female sex, absent social support, and COVID-19-related post-traumatic stress disorder [22], signifying a greater emotional reactivity and stress reaction in women. Furthermore, previous cardiovascular studies suggested that marital and family stress is a strong risk factor for the development of atherosclerosis in women [23]. Accordingly, the significance of psychosocial interventions, with a focus on managing family-related stress in women, should be emphasized [24]. For post-COVID-19 fatigue and the contributing psychological factors reported to a physician, one must consider the potential for sex-related differences in how, and if, symptoms are reported and perceived, which might affect outcomes of interventions and studies.

Biological sex is also a relevant factor for depressive disorders and short- and long-term COVID-19 outcomes [25]. Depression is a psychiatric condition comprised of alterations in behavior, affect, and mood regulators [26]. Women have a greater frequency of risk factors that tend to amplify during a pandemic, including pre-existent depressive and anxiety disorders, consistent environmental stress, and domestic violence [27]. A great risk for neurological and psychiatric illness in the 6 months post-COVID-19 infection might occur, especially in patients who had severe COVID-19 symptoms [9,28]. For women in homemaker roles, the additional caretaking associated with childcare, home education, and caring for sick family members might result in greater mental distress and depression. Furthermore, working women with families might have less time to dedicate to work or professional development, which could lead to social discrimination. In single women who live alone, isolation and the decrease in social relations, together with concern over the potential economic crisis from the pandemic, may further expediate depression. 

Stress, depression, and anxiety might also lead to lifestyle changes, such as unhealthy diets, sedentary behaviors, and eating or drinking to handle stress. These lifestyle changes affect men and women differently, with women more likely to develop a reliance on food craving to handle stress. Food craving is described as a greater consumption of fat- and sugar-rich foods that are highly associated with obesity, which is a well-known risk factor for COVID-19 and greater inflammation [27].

In addition, generalized pain (myalgia) is a common symptom endured by acute COVID-19 patients, with an estimated prevalence of 15% to 20% [29,30]. A multicenter study by Fernández-de-las-Peñas et al. [31] found that the occurrence of post-COVID-19 musculoskeletal pain 8 months post-discharge was 45.1%. Furthermore, the prevalence of *de novo* post-COVID-19 musculoskeletal pain was 74.9%, potentially associated with the presence of previous symptoms. The presence of myalgia and headache, a history of musculoskeletal pain, and female sex as COVID-19-associated symptoms as well as the duration of hospital stay were all significant risk factors. However, it is relevant to note that women also have a greater perception of pain than men [32].

### 2.2. Inflammation

Stress, anxiety, depression, pain, and post-COVID-19 fatigue seem to be co-occurring and potentially codependent; they also share the common risk factor of inflammation. COVID-19 can cause profound immune response alterations, which might be influenced by the sex of the patient [33]. Because many inflammatory disorders, which are common in women [34], are also intensified by psychological stress [35,36], sex differences in cytokine responses, such as interleukin-6 (IL-6), to stress may represent an important underlying mechanism [37]. IL-6 is a cytokine released by the immune system to help fight disease, but IL-6 also causes inflammation and has been associated with fatigue, stress, sleep [38], depression, pain, and mood disorders [39,40,41,42]. IL-6 is engaged in the development of fatigue in both autoimmune and non-autoimmune diseases. It is secreted during acute and chronic inflammatory responses by many cells, including endothelial, immune, and muscle cells. Thus, a major finding by Durstenfeld et al. [38] was that IL-6 was elevated among most of those with post-acute sequelae of COVID-19, with higher levels in women and in those with the central sensitization phenotype (e.g., fatigue, pain, depression, and anxiety) compared with the cardiopulmonary phenotype. In a study conducted by the Mayo Clinic Post-COVID-19 Care Clinic [39], several different clinical phenotypes were also observed. Fatigue dominance was the most frequent presentation and was linked with elevated IL-6 levels and female sex. Additionally, the majority of patients had a significant rise in IL-6. These patients—57% of the cohort—had high IL-6 levels up to three months after being infected with COVID-19. Jankoord et al. [43] found that women concomitantly displayed greater stalk median eminence (SME) content of IL-6 and greater HPA responsiveness to stress, thereby implying that IL-6 release from the SME is an integral factor contributing to enhanced stress responsiveness in women. Furthermore, their results indicated an association between IL-6 and adrenocorticotropic hormone (ACTH) release and a sex difference in this association. However, further research is required to clarify the role of IL-6 on post-COVID-19 fatigue in women and men (together and separately) and whether this cytokine might be a valuable biomarker of viral virulence. More work investigating the potential therapeutic use of blocking IL-6 might offer knowledge about controlling persistent viral infections and/or enduring post-infection fatigue.

Sex-specific differences in the regulation of the hormonal stress and inflammatory responses might also contribute to sexual dimorphism in COVID-19 [44]. This is reinforced by the fact that some diseases with an autoimmune context, such as rheumatoid arthritis, systemic lupus erythematosus, and Grave’s disease, show a predominance in women [45]. Sex hormones can regulate the immune reaction during infections; for example, higher testosterone levels have been linked with less antibody production [46]. In dealing with viral infections, the immune systems of women act differently than men, which create a stronger immune response and more efficient viral clearance [47]. In general, antibody production levels are higher in women than in men and this production tends to last longer [48]. Differences in the immune response of women can be related to sex hormones and factors related to the X chromosome. Estrogen modulates pro-inflammatory responses, and immune regulatory genes are located on chromosome X [49]. Thus, it can be assumed that the cytokine storm linked to immune dysregulation might occur less in women. However, concrete evidence of immunological differences in men and women with SARS-CoV-2 infection is still elusive. Furthermore, from a biological perspective, Stewart et al. [50] proposed the asymmetry in risk and outcomes between the sexes and an overlap of long COVID symptoms [51] with perimenopause and menopause [52], indicating that sex hormone differences are a relevant target for future study. Furthermore, the higher prevalence of long COVID in women ≤ 50 years old [13] might be an important factor as the mean age of natural menopause is 51 years [53].

### 2.3. Pre-Existing Conditions

As described by Rudroff et al. [9] the physical and mental capacity (e.g., pre-existing conditions) of the individual are important elements of fatigue in COVID-19 survivors. Pre-existing conditions, such as asthma, which is more common in women than in men, have been reported to further increase the risk of developing so-called long COVID [54,55]. As indicated by van Herck et al. [55], fatigue is highly prevalent in patients with asthma. Gebhard et al. [20] also reported a link between a pre-existing diagnosis of mental illness and the occurrence of post-COVID-19 syndrome in women, but not in men. This sex-specific association might be related to the higher incidence of mental illness in women. Additionally, preliminary data indicate that dysfunctional immune cells with an autoimmune phenotype are present in patients with post-COVID-19 syndrome, especially in those with permanent neurological symptoms [56,57]. Fatigue is also a common symptom in other comorbidities (compounding conditions) such as diabetes, cancer, cardiovascular disease, congestive heart failure, hypertension, chronic kidney disease, and chronic obstructive pulmonary disease (COPD). Thus, these pre-existing conditions might contribute to post-COVID-19 fatigue, especially in women.

## 3. Conclusions and Future Directions

Fatigue has been identified as one of the most common debilitating symptoms reported by post-COVID-19 patients. Women are at a higher risk for developing long-term post-COVID-19 fatigue and fatigue-related symptoms such as stress, anxiety, depression, and pain than men. In this perspective paper, we described factors that contribute to post-COVID-19 fatigue in women. However, we recognize that this list is far from complete; our model is hypothetical and further research is required to clarify all mechanisms of post-COVID-19 fatigue in women and to validate our proposed theory. To this end, research studies should focus on distinctly defined outcome variables that contribute to fatigue.

It is important to note that fatigue is a symptom that can only be assessed by self-report [58,59,60]. Clinically, fatigue is most often measured with questionnaires that require respondents to estimate their capacity to perform several physical, cognitive, and psychosocial tasks, usually in reference to a pre-disease or condition state. These retrospective estimates provide a measure of *perceived fatigability*. Questionnaires are also used for the estimation of fatigue-related factors, such as stress, anxiety, pain, and depression. Thus, in addition to perceived fatigability, information on performance fatigability [9,61] (magnitude or rate of change in performance) in post-COVID-19 women is required. For example, a pilot study by Workman et al. [62] indicated that perceptions of fatigue, but not performance fatigability, play a dominant role in post-COVID-19 patients who suffer from persistent fatigue symptoms. Thus, including both measures in fatigue studies can help disambiguate these different aspects of fatigue. In addition, long-term longitudinal studies are needed to fully understand the sex-related pathophysiology of fatigue and the effects of pharmacological and non-pharmacological treatments associated with post-COVID-19 fatigue. Furthermore, how vaccination and new variants of SARS-CoV-2 influence sex differences in post-COVID-19 fatigue symptoms may be an important topic to investigate. These studies are necessary to understand the natural trajectory of post-COVID-19 fatigue in women and men and to apply targeted treatment strategies.

## References

[1] Coronavirus Disease (COVID-19)—World Health Organization. https://www.who.int/emergencies/diseases/novel-coronavirus-2019.

[2] Carfi A., Bernabei R., Landi F. (2020). Persistent symptoms in patients after acute COVID-19. JAMA.

[3] Tenforde M.W., Kim S.S., Billig Rose E., Shapiro N.I., Files C.D., Gibbs K.W., Erickson H.L., Steingrub J.S., Smithline H.A., Gong M.N. (2020). Symptom Duration and Risk Factors for Delayed Return to Usual Health Among Outpatients with COVID-19 in a Multistate Health Care Systems Network—United States, March–June 2020. Centers for Disease Control and Prevention. Morb. Mortal. Wkly. Rep..

[4] Huang C., Wang Y., Li X., Ren L., Zhao J., Hu Y., Zhang L., Fan G., Xu J., Gu X. (2020). Clinical features of patients infected with 2019 novel coronavirus in Wuhan, China. Lancet.

[5] Wang D., Hu B., Hu C., Zhu F., Liu X., Zhang J., Wang B., Xiang H., Cheng Z., Xiong Y. (2020). Clinical characteristics of 138 hospitalized patients with 2019 novel coronavirus–infected pneumonia in Wuhan, China. JAMA.

[6] Xu X.-W., Wu X.-X., Jiang X.-G., Xu K.-J., Ying L.-J., Ma C.-L., Li S.-B., Wang H.-Y., Zhang S., Gao H.-N. (2020). Clinical findings in a group of patients infected with the 2019 novel coronavirus (SARS-Cov-2) outside of Wuhan, China: Retrospective case series. BMJ.

[7] Townsend L., Dyer A.H., Jones K., Dunne J., Mooney A., Gaffney F., O’Connor L., Leavy D., O’Brien K., Dowds J. (2020). Persistent fatigue following SARS-CoV-2 infection is common and independent of severity of initial infection. PLoS ONE.

[8] Rudroff T., Fietsam A.C., Deters J.R., Bryant A.D., Kamholz J. (2020). Post-COVID-19 Fatigue: Potential Contributing Factors. Brain Sci..

[9] Huang C., Huang L., Wang Y., Li X., Ren L., Gu X., Kang L., Guo L., Liu M., Zhou X. (2021). 6-month consequences of COVID-10 in patients discharged from hospital: A cohort study. Lancet.

[10] Fernández-De-Las-Peñas C., Martín-Guerrero J.D., Pellicer-Valero J., Navarro-Pardo E., Gómez-Mayordomo V., Cuadrado M.L., Arias-Navalón J.A., Cigarán-Méndez M., Hernández-Barrera V., Arendt-Nielsen L. (2022). Female Sex Is a Risk Factor Associated with Long-Term Post-COVID Related-Symptoms but Not with COVID-19 Symptoms: The LONG-COVID-EXP-CM Multicenter Study. J. Clin. Med..

[11] Ceban F., Ling S., Lui L.M., Lee Y., Gill H., Teopiz K.M., Rodrigues N.B., Subramaniapillai M., Di Vincenzo J.D., Cao B. (2022). Fatigue and cognitive impairment in Post-COVID-19 Syndrome: A systematic review and meta-analysis. Brain Behav. Immun..

[12] Bechmann N., Barthel A., Schedl A., Herzig S., Varga Z., Gebhard C. (2022). Sexual dimorphism in COVID-19: Potential clinical and public health implications. Lancet Diabetes Endocrinol..

[13] Sigfrid L., Drake T.M., Pauley E., Jesudason E.C., Olliaro O., Lim W.S., Gillesen A., Berry C., Lowe D.J., McPeake J. (2021). Long Covid in adults discharged from UK hospitals after Covid-19: A prospective, multicentre cohort study using the ISARIC WHO Clinical Characterization Protocol. Lancet Reg. Health—Eur..

[14] Office for National Statistics Which Occupations Have the Highest Potential Exposure to the Coronavirus (COVID-19)?. https://www.ons.gov.uk/employmentandlabourmarket/peopleinwork/employmentandemployeetypes/articles/whichoccupationshavethehighestpotentialexposuretothecoronaviruscovid19/2020-05-11.

[15] Takahashi T., Ellingson M.K., Wong P., Israelow B., Lucas C., Klein J., Silva J., Mao T., Oh J.E., Tokuyama M. (2020). Sex differences in immune responses that underlie COVID-19 disease outcomes. Nature.

[16] Office for National Statistics COVID-19 Schools Infection Survey Round 2, England: December 2020. https://www.ons.gov.uk/peoplepopulationandcommunity/healthandsocialcare/conditionsanddiseases/bulletins/covid19schoolsinfectionsurveyround2england/december2020.

[17] Morgul E., Bener A., Atak M., Akyel S., Aktas S., Bhugra D., Ventriglio A., Jordan T.R. (2020). COVID-19 pandemic and psychological fatigue in Turkey. Int. J. Soc. Psychiatry.

[18] Satici B., Gocet-Tekin E., Deniz M.E., Satici S.A. (2020). Adaptation of the fear of COVID-19 scale: Its association with psychological distress and life satisfaction in Turkey. Int. J. Ment. Health Addict..

[19] Brooks S.K., Webster R.K., Smith L.E., Woodland L., Wessely S., Greenberg N., Rubin G.J. (2020). The psychological impact of quarantine and how to reduce it: Rapid review of the evidence. Lancet.

[20] Gebhard C.E., Suetsch C., Bengs S., Deforth N., Buehler K.P., Hamouda N., Meisel A., Schuepbach R.A., Zinkernagel A.S., Brugger S.D. (2021). Sex- and Gender-specific Risk Factors of Post-COVID-19 Syndrome: A Population-based Cohort Study in Switzerland. medRxiv.

[21] Lee A.M., Wong J.G., McAlonan G.M., Cheung V., Cheung C., Sham P.C., Chu C.-M., Wong P.-C., Tsang K.W., Chua S.E. (2007). Stress and psychological distress among SARS survivors 1 year after the outbreak. Can. J. Psychiatry.

[22] Cai X., Hu X., Ekumi I.O., Wang J., An Y., Li Z., Yuan B. (2020). Psychological Distress and Its Correlates Among COVID- 19 Survivors During Early Convalescence Across Age Groups. Am. J. Geriatr. Psychiatry.

[23] Orth-Gomér K., Schneiderman N., Wang H.X., Walldin C., Blom M., Jernberg T. (2009). Stress reduction prolongs life in women with coronary disease: The Stockholm Women’s Intervention Trial for Coronary Heart Disease (SWITCHD). Circ. Cardiovasc. Qual. Outcomes.

[24] Wang H.-X., Leineweber C., Kirkeeide R., Svane B., Schenck-Gustafsson K., Theorell T., Orth-Gomér K. (2007). Psychosocial stress and atherosclerosis: Family and work stress accelerate progression of coronary disease in women. The Stockholm Female Coronary Angiography Study. J. Intern. Med..

[25] Almeida M., Shrestha A.D., Stojanac D., Miller L.J. (2020). The impact of the COVID-19 pandemic on women’s mental health. Arch. Womens Ment. Health.

[26] Bucciarelli V., Nasi M., Bianco F., Seferovic J., Ivkovic V., Gallina S., Mattioli A.V. (2022). Depression pandemic and cardiovascular risk in the COVID-19 era and long COVID syndrome: Gender makes a difference. Trend Cardiovasc. Med..

[27] Mattioli A.V., Pinti M., Farinetti A., Nasi M. (2020). Obesity risk during collective quarantine for the COVID-19 epidemic. Obes. Med..

[28] Taquet M., Geddes J.R., Husain M., Luciano S., Harrison P.J. (2021). 6-month neurological and psychiatric outcomes in 236 379 survivors of COVID-19: A retrospective cohort study using electronic health records. Lancet Psychiatry.

[29] Abdullahi A., Candan S.A., Abba M.A., Bello A.H., Alshehri M.A., Afamefuna Victor E., Umar N.A., Kundakci B. (2020). Neurological and musculoskeletal features of COVID-19: A systematic review and meta-analysis. Front. Neurol..

[30] Ciaffi J., Meliconi R., Ruscitti P., Berardicurti O., Giacomelli R., Ursini F. (2020). Rheumatic manifestations of COVID-19: A systematic review and metaanalysis. BMC Rheumatol..

[31] Fernández-de-las-Peñas C., de-la-Llave-Rincon A.I., Ortega-Santiago R., Ambite-Quesada S., Gomez-Mayordormo V., Cuadrado M.L., Hernández-Barrera V., Martín-Guerrero J.D., Pellicer-Valero J.O., Arendt-Nielsen L. (2022). Prevalence and risk factors of musculoskeletal pain symptoms as long-term post-COVID sequelae in hospitalized COVID-19 survivors: A multicenter study. Pain.

[32] Barsky A.J., Peekna H.M., Borus J.F. (2001). Somatic symptom in women and men. J. Gen. Intern. Med..

[33] Rabaan A.A., Al-Ahmed S.H., Garout M.A., Al-Quaaneh A.M., Sule A.A., Tirupathi R., Al Mutair A., Alhumaid S., Hasan A., Dhawan M. (2021). Diverse immunological factors influencing pathogenesis in patients with COVID-19: A review on viral Dissemination, Immunotherapeutic options to counter cytokine storm and inflammatory responses. Pathogens.

[34] Whitacre C.C., Reingold S.C., O’Looney P.A. (1999). A gender gap in autoimmunity. Science.

[35] Kimyai-Asadi A., Usman A. (2001). The role of psychological stress in skin disease. J. Cutan. Med. Surg..

[36] Lechin F., van der D.B., Jackubowicz D., Camero R.E., Lechin S., Villa S., Reinfeld B., Lechin M.E. (1987). Role of stress in the exacerbation of chronic illness: Effects of clonidine administration on blood pressure and plasma norepinephrine, cortisol, growth hormone and prolactin concentrations. Psychoneuroendocrinology.

[37] Edwards K.M., Burns V.E., Ring C., Carroll D. (2006). Sex difference in the interleukin-6 response to acute psychological stress. Biol. Psychol..

[38] Durstenfeld M.S., Peluso M.J., Kelly J.D., Win S., Swaminathan S., Li D., Arechiga V.M., Zepeda V., Sun K., Shao S. (2021). Role of antibodies, inflammatory markers, and echocardiographic findings in post-acute cardiopulmonary symptoms after SARS-CoV-2 infection. medRxiv.

[39] Ganesh R., Grach S.L., Ghosh A.K., Bierle D.M., Salonen B.R., Collins N.M., Joshi A.Y., Boeder N.D., Anstine C.V., Mueller M.R. (2022). The Female-Predominant Persistent Immune Dysregulation of the Post-COVID Syndrome. Mayo Clin. Proc..

[40] Rohleder N., Aringer M., Boentert M. (2012). Role of interleukin-6 in stress, sleep, and fatigue. Ann. N. Y. Acad. Sci..

[41] Grygiel-Górniak B., Puszczewicz M. (2015). Fatigue and interleukin-6—A multi-faceted relationship. Reumatologia.

[42] Bossola M., Di Stasio E., Giungi S., Rosa F., Tazza L. (2015). Fatigue is associated with serum interleukin-6 levels and symptoms of depression in patients on chronic hemodialysis. J. Pain Symptom Manag..

[43] Jankord R., Turk J.R., Schadt J.C., Casati J., Ganjam V.K., Price E.M., Keisler D.H., Laughlin M.H. (2007). Sex difference between interleukin-6 and stress. Endocrinology.

[44] Veldhuis J.D., Sharma A., Roelfsema F. (2013). Age-dependent and gender dependent regulation of hypothalamic-adrenocorticotropic-adrenal axis. Endocrinol. Metab. Clin. N. Am..

[45] Amur S., Parekh A., Mummaneni P. (2012). Sex differences and genomics in autoimmune diseases. J. Autoimmun..

[46] Acharya Y., Pant S., Gyanwali P., Dangal G., Karki P., Bista N.R., Tandan M. (2020). Gender disaggregation in COVID-19 and increased male susceptibility. J. Nepal Health Res. Counc..

[47] Falahi S., Kenarkoohi A. (2021). Sex and differences in the outcome of patients with COVI-19. J. Med. Virol..

[48] Jin J.-M., Bai P., He W., Wu F., Liu X.F., Han D.M., Liu S., Yang J.-K. (2020). Gender differences in patients with COVID-19: Focus on severity and mortality. Front. Public Health.

[49] Conti P., Younes A. (2020). Coronavirus COV-19/SARS-CoV-2 affects women less than men: Clinical response to viral infection. J. Biol. Regul. Homeost. Agents.

[50] Stewart S., Newson L., Briggs T.A., Grammatopoulos D., Young L., Gill P. (2021). Long COVID Risk—A signal to address sex hormones and women’s health. Lancet Reg. Health—Eur..

[51] Nalbandian A., Sehgal K., Gupta A., Madhavan M.V., McGroder C., Stevens J.S., Cook J.R., Nordvig A.S., Shalev D., Sehrawat T.S. (2021). Post-acute COVID-19 syndrome. Nat. Med..

[52] Sudre C.H., Murray B., Varsavsky T., Graham M.S., Penfold R.S., Bowyer R.C., Pujol J.C., Klaser K., Antonelli M., Canas L.S. (2021). Attributes and predictors of long COVID. Nat. Med..

[53] National Institute for Health and Care Excellence NICE|CKS|Health Topics A to Z|Menopause. Clinical Knowledge Summaries. https://cks.nice.org.uk/topics/menopause/.

[54] Leynaert B., Sunyer J., Garcia-Esteban R., Svanes C., Jarvis D., Cerveri I., Dratva J., Gislason T., Heinrich J., Janson C. (2012). Gender differences in prevalence, diagnosis and incidence of allergic and non-allergic asthma: A population-based cohort. Thorax.

[55] Van Herck M., Spruit M.A., Burtin C., Djamin R., Antons J. (2018). Fatigue is Highly Prevalent in Patients with Asthma and Contribute to the Burden of Disease. J. Clin. Med..

[56] Natelson B.H., Haghighi M.H., Ponzio N.M. (2002). Evidence for the Presence of Immune Dysfunction in Chronic Fatigue Syndrome. Clin. Vaccine Immunol..

[57] Song E., Bartley C.M., Chow R.D., Ngo T.T., Jiang R., Zamecnik C.R., Dandekar R., Loudermilk R.P., Dai Y., Liu F. (2021). Divergent and self-reactive immune responses in the CNS of COVID-19 patients with neurological symptoms. Cell Rep. Med..

[58] Penner I.K., Paul F. (2017). Fatigue as a symptom or comorbidity of neurological diseases. Nat. Rev. Neurol..

[59] Kuppuswamy A. (2017). The fatigue conundrum. Brain.

[60] Reitberg M.B., van Wegen E.E.H., Kwakkel G. (2010). Measuring fatigue in patients with multiple sclerosis: Reproducibility, responsiveness and concurrent validity of three Dutch self-report questionnaires. Disabil. Rehabil..

[61] Rudroff T., Kindred J.H., Ketelhut N.B. (2016). Fatigue in Multiple Sclerosis: Misconceptions and Future Research Directions. Front. Neurol..

[62] Workman C.D., Ponto L.L., Kamholz J., Bryant A.D., Rudroff T. Transcranial Direct Current Stimulation and Post-COVID-19-Fatigue. Proceedings of the 4th Brain Stimulation Conference.

